# The Application of CAR-T Cells in Haematological Malignancies

**DOI:** 10.1007/s00005-020-00599-x

**Published:** 2020-11-06

**Authors:** Katarzyna Skorka, Katarzyna Ostapinska, Aneta Malesa, Krzysztof Giannopoulos

**Affiliations:** Department of Experimental Hematooncology, Medical University of Lublin, Chodzki 1, 20-093 Lublin, Poland

**Keywords:** Chimeric antigen receptor, Chimeric antigen receptor T-cell, Lymphoblastic leukaemia, Diffuse large B-cell lymphoma, Chronic lymphocytic leukaemia, Multiple myeloma

## Abstract

Chimeric antigen receptor (CAR)-T cells (CART) remain one of the most advanced and promising forms of adoptive T-cell immunotherapy. CART represent autologous, genetically engineered T lymphocytes expressing CAR, i.e. fusion proteins that combine components and features of T cells as well as antibodies providing their more effective and direct anti-tumour effect. The technology of CART construction is highly advanced in vitro and every element of their structure influence their mechanism of action in vivo. Patients with haematological malignancies are faced with the possibility of disease relapse after the implementation of conventional chemo-immunotherapy. Since the most preferable result of therapy is a partial or complete remission, cancer treatment regimens are constantly being improved and customized to individual patients. This individualization could be ensured by CART therapy. This paper characterized CART strategy in details in terms of their structure, generations, mechanism of action and published the results of clinical trials in haematological malignancies including acute lymphoblastic leukaemia, diffuse large B-cell lymphoma, chronic lymphocytic leukaemia and multiple myeloma.

## Introduction

Adoptive T-cell immunotherapy represents a novel approach to treat malignancies by modifying T lymphocytes of the patient to recognize and eliminate cancer cells more effectively. This kind of strategy involves methods with the use of genetic engineering to introduce antigen-specific receptors on the surface of grafted T cells, which are then infused into the bloodstream. Consequently, it enables to specifically target malignant cells, which results in better therapy outcomes and potentially less severe adverse effects than in other treatments.

Currently, there are three approaches to adoptive T-cell therapies developed to treat cancer, including T-cell receptor (TCR) modified T cells, tumour-infiltrating lymphocytes and chimeric antigen receptor (CAR) T cells (CART). Amongst these methods, CART remain one of the most advanced and promising form of adoptive T-cell immunotherapy. CART represents autologous, genetically engineered T lymphocytes expressing CAR aside from their natural TCRs. CARs are fusion proteins that combine components and features of T cells as well as antibodies thereby their anti-tumour activity is more effective. Since CART are equipped in costimulatory molecules they can be activated independently on antigen-presenting cells (Gross et al. 1989; June et al. 2018; Rohaan et al. 2019).

Patients with haematological malignancies are faced with the possibility of disease relapse after the implementation of conventional chemo-immunotherapy. Since the most preferable result of therapy is a partial or complete remission, cancer treatment regimens are constantly being improved and customized to individual patients. This individualization could be ensured by CART therapy. In the current paper, we characterized the mechanism of action of CART and their application in multiple clinical trials performed in haematological malignancies (June et al. 2018; Rohaan et al. 2019).

## Structure of CART

The general structure of CAR comprises of an antigen-binding domain isolated from the antibody and an activating domain derived from the TCR. The main concept behind the making of this synthetic T-cell construct was to combine the antibody specificity properties with regular T-cell functions, such as proliferation, cytokine production, and elimination of targeted cells (Gross et al. 1989). CAR expression enables modified T cells to acquire more advanced and directed anti-tumour properties (Sun et al. 2018).

**Fig. 1:**
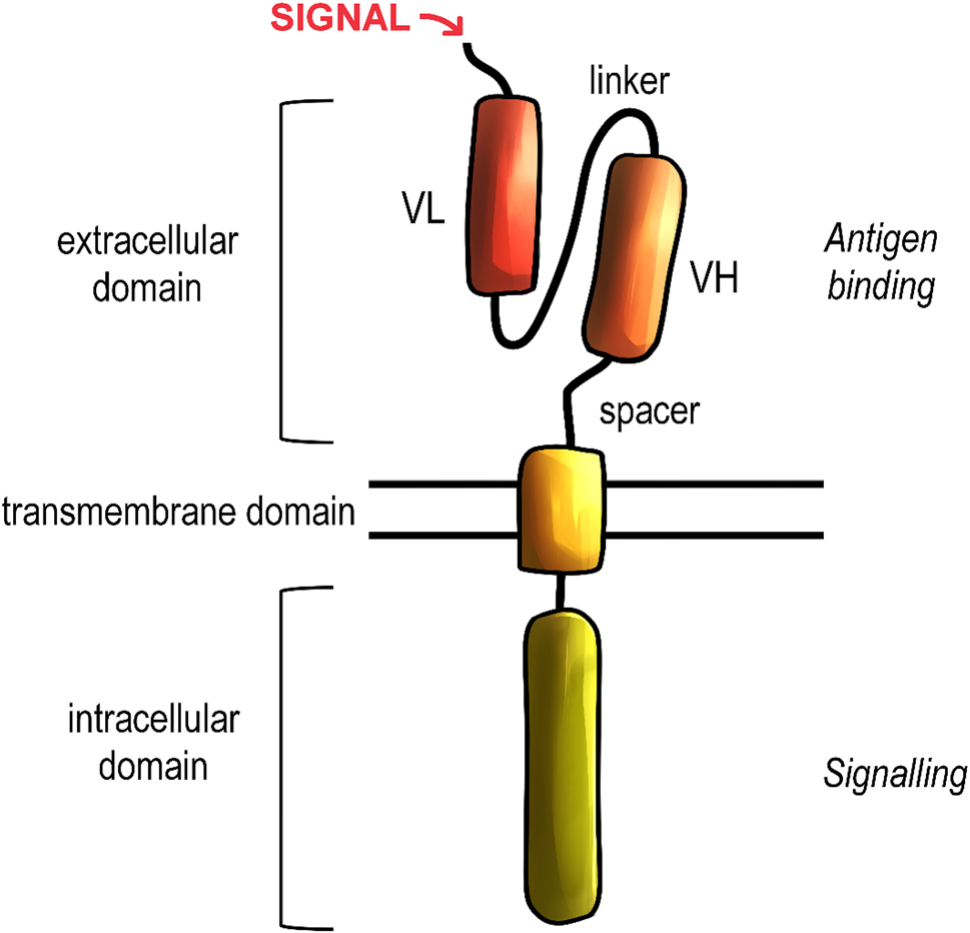
General structure of the chimeric antigen receptor (CAR). CARs consist of an extracellular, transmembrane and intracellular domains. The extracellular domain is responsible for antigen binding and it includes the single-chain variable fragment, derived from the antibody domains, precisely variable heavy (VH) and light (VL). The domains are connected together via linker and anchored in the transmembrane domain by a spacer. The transmembrane domain is responsible for the stabilization of CAR. The intracellular domains are derived from the T-cell receptor and are responsible for inducing the cell response after the antigen recognition

CAR consists of the extracellular domain, which specifies a CART target, the spacer domain followed by the transmembrane domain, and then the intracellular domain (Fig. 1). The intracellular part of the receptor is formed by multiple signalling domains. Each of those domains has a significant impact on the safety of engineered T cells, the CAR expression on the T-cell membrane, and thus the efficacy of therapy (Makita et al. 2017).

The binding domain, which is located on the extracellular part of CAR, can recognize surface antigens expressed on target cells (Lee and Kim 2019). Effectiveness of antigen recognition depends on the binding affinity determined by the ectodomain of CARs. While essential molecules recognized by regular TCRs are proteins, the introduction of CARs extended the range of recognized targets to not only proteins but also carbohydrates and glycolipids (Irving et al. 2017). The binding domain is most commonly derived from the single-chain variable fragments (scFv) of antibodies (June et al. 2018). The scFv is formed by light and heavy chains fused with a short, flexible linker and it is derived from an antigen-binding region on a monoclonal antibody (Zhang et al. 2017; Dreger et al. 2019). This segment of the antibody directs the T-cell-antigen-binding process with the high affinity and indirectly initiates the T-cell activation. In contrast with TCRs, CARs can recognize the antigen independently of major histocompatibility complex (MHC) restrictions (Muhammad et al. 2017). This modification enables targeting malignancies that are characterized by downregulation of MHC components, including the human leukocyte antigen class I molecules, and to bypass the impaired antigen processing in the immune-evading tumour microenvironment (Dotti et al. 2014).

The spacer domain, which is also called a hinge region, is a linker between the extracellular and transmembrane domains. In the current CAR models, sequences used for the expression of spacer domains can be adopted from flexible regions of proteins expressed on T cells, including CD8 or CD28. However, the most prevalent solution is using the Fc region presented in IgG1 and IgG4 immunoglobulins (Labanieh et al. 2018). Hinge regions derived from clusters of differentiation are built from approximately 40 amino acids (aa) and do not form any specific secondary structures. Fc-arranged domains are about 200 aa long and their organization is characterized by tertiary structures (Lee and Kim 2019). The hinge domain prevents the flexibility of the exogenous part of CAR to maintain a proper level of target recognition (Elahi et al. 2018). The length of this domain partially defines CAR-binding capability, and thus the antigen-of-interest recognition (Xu et al. 2018). The spacer is followed by the transmembrane domain, which is a part that combines extra- and intracellular components of CAR (Gilham et al. 2012).

Stability is another crucial property of CARs and it can be preserved due to the presence of a hydrophobic alpha helix that spans the cellular membrane (Zhang et al. 2017). The non-polar amino acid residues form its secondary alpha-helical structure. This region of CAR is called the transmembrane domain and it is the only part of CAR exposed to the hydrophobic environment. Transmembrane domains are frequently derived from CD4, CD8α*,* or CD28 molecules, similarly to the spacer domains (Lee and Kim 2019). CART characterised by CD3ζ-derived transmembrane domain demonstrate the ability to form complexes with endogenous antigens. The application of CD3ζ transmembrane domain can affect the target specificity of CARs (Bagley et al. 2010).

The signalling domain is an endogenous, functional part of CAR. Its activation is responsible for CAR-mediated immune responses, such as cytokine release, cytolysis or maintaining proper T-cell proliferation (Vairy et al. 2018). The number and properties of signalling domains are specified by the generation of CART. The fundamental component of the signalling domain is the CD3ζ chain, which provides the activating signal in engineered T cells (Hombach et al. 2001; Yeku and Brentjens 2016). The activating signal is conducted by three immunoreceptor tyrosine-based activation motifs (ITAMs). The activation signal is initiated after the antigen recognition through the phosphorylation of ITAMs, leading to the activation of the signalling cascade in the lymphocyte cytoplasm (Love and Hayes 2010).

**Fig. 2:**
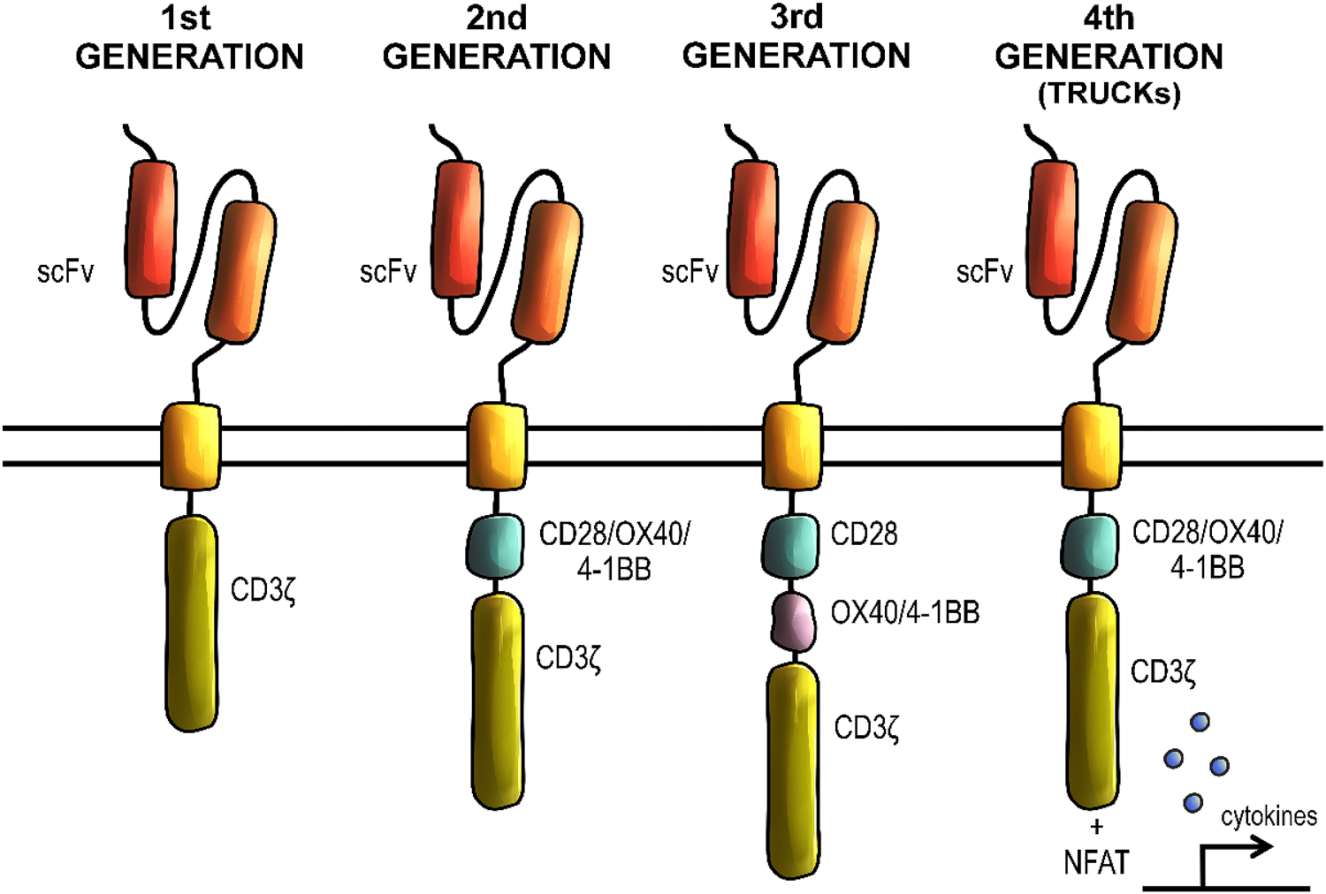
Generations of CARTs. The first generation of CAR-T cells is used as a template to construct later generations and its signaling is based on the presence of the intracellular CD3ζ domain. The second generation CAR-T cells (CARTs) incorporate a costimulatory domain, most often CD28. Third generation CARTs incorporate additional costimulatory domains, such as CD28, ICOS, 4-1BB or OX40. Fourth generation CARTs, also called TRUCKs, are based off second generation CARTs with an additional gene cassette, which induces cytokine expression. *scFv* single-chain variable fragment, *NFAT* nuclear factor of activated T cells

CART are divided according to the character of their signalling domains. The first-generation CART have only one stimulatory domain that is usually derived from the CD3ζ chain or FcRc (Lee and Kim 2019). The second-generation CART extended the basic format of CAR with a single co-stimulatory domain and, subsequently, the third generation comprised two additional co-stimulatory domains (Sadelain et al. 2013). The fourth generation, which contains only one co-stimulatory domain, is specifically engineered with the nuclear factor of the activated T-cell (NFAT) to direct the cell to express transgenic products, such as cytokines (Chmielewski and Abken 2015). So far, four generations of CART have been fully developed, although there have been studies approaching a concept of next-generation, or fifth-generation CART (Fig. 2) (Muhammad et al. 2017).

## Generations of CART

First-generation CART are used as a template for further modifications of domains (Bridgeman et al. 2010). The purpose of modifying single subunits of CART is to improve their efficacy by enhancing their signalling, inducing specific cytokines release, and thus stimulating the induced T-cell proliferation and immune response (Mata and Gottschalk 2019). The function of CART in most often driven by a direct T-cell interaction with malignant cells, as well as the release of various interleukins (IL) in the tumour microenvironment. Additionally, specific stimulatory components amongst CART generations can induce different cytokine responses. CART manipulations and improvements aim to enhance their safety and reduce related toxicities, minimizing the death rate among the patients (Zhu et al. 2016).

The architecture of the first generation of CART is rather basic since their signalling domain comprises only of the CD3ζ chain (Kowolik et al. 2006). The target domain of first-generation CART can include either scFv or Fab fragments, but scFv domains are predominant (Sadelain et al. 2013; Elahi et al. 2018). Since first-generation CART do not incorporate any additional signalling domains, they are only able to mediate optimal cellular activation via dimerization (Bagley et al. 2010). This results in insufficient interleukin production and, overall, a not desired outcome, as reported in many clinical trials (Brocker 2000; Till et al. 2008; Savoldo et al. 2011; Ramos et al. 2014). The low potential of activation and proliferation of T cells is because the activation is initiated by antigen-dependent signalling. It remains fully independent of co-stimulation, which requires additional domains, but it also results in higher efficacy (Kowolik et al. 2006).

The second generation of CART, most often used in the clinic, encompass co-stimulatory domain in addition to the first-generation properties. This combination allows the activation of a secondary signalling pathway, resulting in a higher overall efficacy (Elahi et al. 2018). Consequently, the fundamental improvement characteristic of second-generation CART is combining the CD3ζ with another co-stimulatory domain, most commonly CD28 or 41BB (CD137), but there are also constructs using OX40 (CD134), CD2, CD27 or inducible T-cell co-stimulator (ICOS) (Song et al. 2012; Hombach et al. 2012). ICOS-based CART are able to increase IL-17A, IL-17F and IL-22 signalling, as well as promote the Th1/Th17 differentiation (Guedan et al. 2014). Including different co-stimulatory domains into the CART structure may result in different functionality of the constructs, as well as their persistence in the bloodstream. It is proven, that in comparison to the first-generation, second-generation CART induce an increased amount of cytokines, notably the interferon (IFN)-γ. A significant change in the second-generation CART activity was acquiring the ability to trigger IL-2 secretion (Hombach et al. 2001). Second-generation CART showed most notably anti-tumour responses in patients with haematologic malignancies (Heyman and Yang 2019).

The third generation of CART are able to activate multiple signalling pathways (Zhang et al. 2017). This generation includes more complex structures characterized by the presence of two co-stimulatory domains. The main principle behind the construction of this subtype of CART was to combine different signalling compounds, mainly CD28 together with 4-1BB, to overcome the limitations present while using only individual domains. Co-stimulation using CD28 enables rapid expansion of CART after the exposure to the antigen, while the co-stimulation of 4-1BB ensures a long-term persistence in the bloodstream, although the exact mechanism responsible for this effect remains unclear (van der Stegen et al. 2015). Additionally, different domains show various potency in activation and proliferation of T cells, as well as the IL-2 production in response to antigen exposure. Unfortunately, despite a promising design of the CD28-4-1BB construct, the application of third-generation CARs represents a clear risk of inadequate signal transmission and excessive cytokine activity, which may lead to both insufficient therapy results and increased toxicity of therapy (Wilkie et al. 2008).

Currently, the most advanced generation of CART are so-called TRUCKS. These constructs merge second-generation CART features with a transgenic product delivery system, enabling an enhanced cytokine expression in the cell microenvironment. This generation of CART features immune-stimulatory cytokines that promote modified T-cell expression and prolong their survival in the immunosuppressive tumour microenvironment. Fourth-generation CART are the only chimeric antigen receptors engineered with the nuclear factor of activated T cells (NFAT). In an ordinary microenvironment, NFAT is responsible for the expression of products essential for the proper cell function, such as cytokines. Activation of TRUCKs leads to the NFAT-mediated induction of cytokine expression. TRUCKs deliver transgenic material directly to the targeted tumour site, thereby avoiding the systemic toxicity. These constructs can also induce a secondary immune response against cells that have not been yet affected. Fourth-generation CART may cause an increased bystander T-cell activity towards cancer cells that do not express antigens recognized by modified T cells. This type of activity enables to eliminate cancer cells that would otherwise remain undetected by antigen-targeting agents. The bystander elimination process is caused by an increased expression of cytokines such as IL-12, IL-15, and IL-18 (Petersen and Krenciute 2019; Zhang et al. 2020). These cytokines also mediate regular TRUCK-induced response to antigen exposure. Manufacturing of TRUCKs requires the introduction of two individual genes into the T-cell construct, one responsible for the expression of CAR and one for the cytokine secretion. The transgenes for the fourth-generation compounds have to be integrated at different genomic sites, as placing them in the same expression region could lead to undesired transactivation. To manufacture TRUCKs, the T cells must undergo double modification. This operation may result in a higher risk of producing non-functional constructs due to excessive modification required for construction of fourth-generation CART (Chmielewski and Abken 2015).

## Manufacturing of CART

Manufacturing of CART is based on collecting T cells from the patient’s blood and genetic modification in order to obtain intended T-cell features. Expression of genes responsible for CAR assembly is possible due to incorporation of aforementioned genes into T-cell via, for instance, viral vectors or transposon systems and then, subsequent induction of expression of CAR coding genes.

This results in the expression of antigen-targeting receptors on the cell membrane. CAR expression enables modified T cells to acquire more advanced and directed anti-tumour properties (Sun et al. 2018). After that, modified T cells can be administered to the patient as therapeutic agents (Wang and Rivière 2016).

**Fig. 3:**
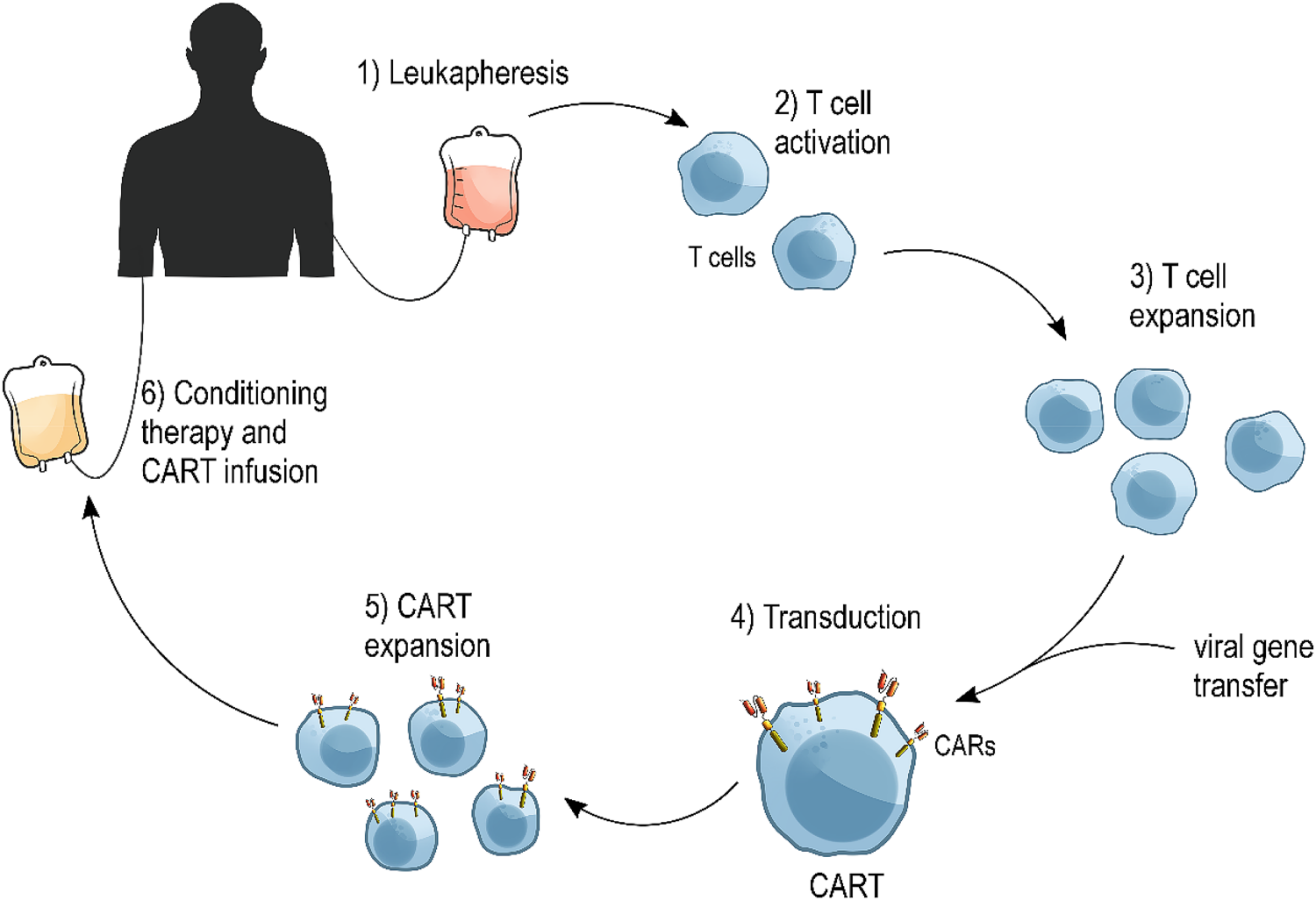
CART production and clinical use. The first step in CART generation is collecting the immune cells from the patient or donor via leukapheresis. T cells are separated from other blood components and their activation and expansion is induced. After that, T cells must undergo a gene transfer process, most often via a viral vector. The gene transfer results in the expression of chimeric antigen receptors (CARs) on the cell surface. Then, the expansion of modified T cells is induced in the bioreactor. After achieving an appropriate volume, modified cells are collected and administered to the patient. Before the infusion, in most cases, patients undergo the lymphodepletion conditioning chemotherapy

Since the first development of CART in 1993, the protocols for generating the constructs are constantly being improved to enhance not only the efficiency, but also the safety of CART therapy. Every step of the process of CART manufacturing undergoes thorough quality control. Despite the differences in generations of CART target antigens, the main manufacturing procedure remains consistent (Fig. 3) (Zhang et al. 2017).

The first step in the generation of CART is leukapheresis (Brown and Adusumilli 2016; Zhang et al. 2017). This step is necessary for obtaining T cells for future modifications. In this procedure, only immune cells are being extracted, while all other blood components are returned to the patient’s or donor’s circulation. Afterwards, the T cells are washed out of leukapheresis buffer (Gomes-Silva and Ramos 2018). Obtained cells have to be activated with the use of beads, covered in anti CD3 or CD28 monoclonal antibodies or with allogeneic dendritic cells. Then activated T cells can be used immediately for genetic modifications in downstream protocol procedures or stored in low temperatures to be used in the future. This enables the collection of the source material from the patient in advance, which may be useful in cases when patients cannot undergo treatment directly after the leukapheresis (Wang and Rivière 2016).

Several methods are enabling the RNA delivery to the patient-derived cells. During this procedure, the vectors introduce genetic material in the form of RNA, which is later reversely transcribed into T-cell DNA. Viral DNA transfers, the most commonly used in CAR-encoding gene delivery, are based on the use of a gamma-retroviral and lentiviral vector. These transfers have a significant threshold to their payload, as retroviral- and lentiviral-derived vectors are capable of delivering ~ 8 kb of genetic material. However, since viral vectors are considered to be potentially oncogenic, prior to the application they need to be extensively tested to exclude the possibility of contacting the replication-competent particles. Using viral vectors in clinical application also requires thorough patient follow-up after the procedure to exclude potential viral insertional mutagenesis in response to the therapy (Gomes-Silva and Ramos 2018).

To prevent the viral vector-associated adverse effects and complications, the transposon-based system was introduced. Inserting the genetic material with this method poses less immunogenicity and enables to insert a larger amount of RNA, up to 100 kb. However, this method requires the use of electroporation, which may permanently damage the patient-derived T cells. Using electroporation to introduce is cheaper, simpler, and potentially safer, but may result in a lowered transduction efficiency, prolonging the CART expansion process and potentially reducing their therapeutic effects. The CART constructs created using the mRNA electroporation have proven to be less persistent, as the modified CAR expression on the cell surface was noted to last about a week. There are some cases when this effect may be desired, for instance in cases of a risk of severe toxicities in response to persistent antigen targeting (Gomes-Silva and Ramos 2018). Still, utilizing non-viral methods to introduce gene constructs into the T-cell is an emerging approach in CART manufacturing. Along with the ongoing progress of expression systems such as CRISPR-Cas9, zinc fingers, TALENs or specific endonucleases, the efficacy of manufacturing CART may be improved. Delivery of genetic material into manufactured T cells results in permanent integration into the genome of patient’s cells (Labanieh et al. 2018).

To make CART active and efficient in their action, the use of some stimulating factors is required. In clinical development, several systems are available for T-cell activation. A range of those consists of the cell-based, bead-based, including antibody-coated magnetic beads nanobeads, and Expamer technology (Frauwirth et al. 2002; Brown and Adusumilli 2016). The process of activation is mostly based on the use of monoclonal antibodies, such as anti-CD3 or anti-CD28. The final procedure for generating CART is T-cell expansion. This phase is accomplished with the use of bioreactors. As the cells divide, CARs are being successfully expressed on the cell surface (Harrison et al. 2019).

When the expansion is finished, the cell culture must be concentrated to a volume that can be applied to the patient as a therapeutic agent. The infusion could be deployed after 48–96 h from completing the lymphodepletion chemotherapy (Turtle et al. 2016). After the cell administration, patients must be closely monitored to track any possible adverse effects and to ensure early mitigation of any complications. Since the adverse effects of therapy appear within the first few days, the hospitalization of patients allows to lower the risk of therapy toxicities and to optimize the treatment outcome. The entire process of CART administration lasts about 3 weeks, with the process of cell preparation being the most time-consuming (Zhao and Cao 2019).

## The Factors Affecting on the Mechanism of Action of CART

Despite the fact that CART show promising clinical outcomes, their efficacy varies depending on many factors, such as the type and availability of target antigen, the type of costimulatory molecules, the structure of extracellular and intracellular domains, the length of the spacer domain, the thorough CAR engineering process and tumour environment. Proper arrangement of therapy is also crucial in achieving satisfactory results of CART-based treatment.

Once the modified T cells are infused into the patient’s blood, they have to reach the site of the target tumour. In contrast to solid tumours, in haematological malignancies, within the moment of infusion, circulating CART achieve their target. In solid tumours, CART encounter multiple barriers before reaching their target, which is crucial to make the therapy efficient (Irving et al. 2017). For instance, physical barriers such as aberrant vasculature appearing in the tumour site can restrict or fully block T-cell entry. Moreover, immunosuppressive specificities of tumour-microenvironment are unfavourable to CART penetrating the neoplasm smoothly (Martinez and Moon 2019). Presumably, CART remain inactive, unless they bind to the corresponding antigen. Some of the CAR constructs induce increased basal activation, being achieved via phosphorylation. Increased basal activation contributes to stronger signal and rapid kinetics in third-generation CARs comprising both CD28 and CD3ζ domains (Salter et al. 2018; Benmebarek et al. 2019). After CAR-antigen connection, the surface domain transmits a signal to the stimulatory domain by itself or accompanied by other, co-stimulatory domains. At this moment, T-cell antigen recognition followed by the cell expansion is required to achieve a therapeutic effect (Ramello et al. 2019).

For optimal activation and efficient proliferation, CART require CD3ζ, co-stimulatory domain-induced signalling, as well as, more importantly, the exogenous signal provided by cytokines that stimulate the immune response. However, those remain inactive in the microenvironment of tumours. To override this obstacle, several strategies aiming at the cytokine delivery have been developed. The delivered cytokines, such as IL-12, IL-21 or IL-18 can affect, for instance, proper T-cell proliferation (Scholler et al. 2015).

Metabolism of CART is driven by the tumour environment and their functional properties, such as costimulatory domains. It is proved, that the presence of 4-1BB domain enhances oxidative metabolism. Conversely, the co-stimulation through the CD28 derived domain effects in more intensive aerobic glycolysis (Irving et al. 2017; Labanieh et al. 2018). The approach of characterization of the CAR signalosome demonstrated that CAR expression promotes the T-cell activation utterly independent of antigen presence (Ramello et al. 2019).

Introducing the conditioning chemotherapy is a crucial step in implementing CART in patients (Levine et al. 2017; Xu et al. 2018). Effective action of T cells requires proper preparation of patients undergoing CART therapy. The initial and crucial step, which provides proper blood-conditions for modified T cells is lymphodepletion, commonly used in the form of conditioning chemotherapy. The main objective of lymphodepletion is to prevent the rejection of transplanted cells by the host (Page et al. 2013). Lymphodepletion is achieved through conditioning chemotherapy or radiotherapy. Researchers have observed a higher response rate in patients who have undergone the conditioning regimen, demonstrating in augmented T-cell proliferation and their persistence in bloodstream (Brentjens et al. 2011).

Treatment efficiency also heavily depends on the type of the targeted antigen. Numerous CAR-based trials focus on different surface antigens, such as CD19, CD20, or CD21 (Watanabe et al. 2014; Wong et al. 2017). Amongst them, the most common therapeutic target is CD19 (Lee et al. 2015). This transmembrane protein is expressed on mature B cells, and thus, it can be classified as a biomarker of tumour cell development in some B-cell malignancies (Wang et al. 2012). Trials focusing on the CD19 antigen have shown to be more effective in comparison to standard chemotherapy. So far, only the CD19-focused CART therapies have been approved by the Food and Drug Administration (FDA). Although CD19 is an attractive target in many malignancies, its efficacy has been mostly proven in acute lymphoblastic leukaemia (ALL) (Fda and Cber 2017; Kymriah 2018).

There has been a constant search for different approaches to improve the efficacy of armored CART (typically modified second generation of CART). One of the ways to increase the effectiveness of CART is to enable them to recognize multiple antigens. This can be achieved by engineering the T cells to target more than one type of cluster of differentiation (CD) molecules present on malignant cells. The major limitation causing therapy failure is an event called antigen loss. Multiple molecular mechanisms are responsible for antigen loss, such as acquired mutations or alternatively spliced *CD19* alleles. This can result in a lack of CD19 surface expression or expression of surface antigens that are not recognized by CART. To overcome the problem of antigen loss, researchers have proposed to target more than one antigen receptors. This can be achieved by four different ways: (1) co-administration, which is a generation of two or more T-cell populations to include in the therapy; (2) bicistronic vector injection, which enables the vectored T-cell to express two different CARs; (3) co-transduction, which remains the most akin to traditional CAR engineering method, because it incorporates simultaneous manipulation of T cells with two different CAR constructs. (4) The last method is to encode two different CARs on the same protein by implementing a single vector. However, despite the fact that multi-targeted CART are a promising link in adoptive T-cell therapy, there are still some questions regarding their safety or efficacy. While the use of multiple targets aims to overcome the target antigen loss phenomenon, this construct is not able to avoid other resistance mechanisms to CART therapy. The other concern stems from the risk of developing severe adverse effects among the patients (Shah et al. 2019).

The type of CART used in therapy also plays a crucial role in proper tumour management. First-generation of CART demonstrated incomplete T-cell activation, resulting in the insufficient secretion of IL-2, which is crucial for the proliferation of T cells (Schmidts and Maus 2018). Since the majority of trials using the first generation, CART did not give desired outcomes, currently, they are thought to be inefficient in clinical use.

Unlike the first generation of CART, which were not capable of inducing a strong cytokine response, the subsequent second generation provides double signalling, thus enhancing of the activating signal. Different variations of co-stimulatory domains have been taken into consideration for clinical use. The CD28 co-stimulatory domain, in addition to the elemental CD3ζ chain, showed outstanding activating properties due to providing the strongest signal for cytokine production and increasing T-cell proliferation without undesired outcomes, such as initiating early cell death (Zhang et al. 2015). The significant role of second-generation CD28 CART has been proven in lymphoma patients, resulting in the improvement of the overall expansion and persistence of modified T cells (Savoldo et al. 2011). It was shown that activation with CD28 provide faster but short-term activation of T cells in contrast to 4-1BB that is responsible for long term activation. The latest studies have shown that second-generation CART with the CD28 domain alone are more efficient than third-generation CART, which are implementing the CD28 domain together with the 4-1BB co-stimulatory component (Ramello et al. 2019).

Third-generation CART have a more complex structure and have the ability to implement multiple signalling into their function. Unfortunately, one of the possible risks associated with their implementation is the possibility of signal leakage and excessive cytokine production (Muhammad et al. 2017).

Since TRUCKs are the most developed constructs used in CART therapy, they enable novel solutions in cancer treatment. The refinements given by this CART generation are still under investigation, but pre-clinical studies (Kueberuwa et al. 2018) have acknowledged their outstanding properties. Due to the ability of transgene protein production, TRUCKs are representing an emerging approach in cancer therapy. This generation of CART is capable of modifying the target cells and this significant improvement is achieved by direct delivery of the transgenic material to the tumour site. Thanks to the transgene product expression, the altered cells acquire the ability to release higher levels of anti-tumour cytokines that enhance the immune response towards malignancies (Chmielewski and Abken 2015). Currently, there is an ongoing clinical trial (NCT03542799) of fourth-generation CARTs in metastatic patients. The study was conducted to evaluate safety and the maximum applicable dose (Chmielewski and Abken 2020). Also, IL-12 TRUCKs have demonstrated efficacy in preclinical models of hematological malignancies, while not requiring the preconditioning (Pegram et al. 2012, 2016).

Moreover, it was shown that that selection of population of T cells for transduction influence effectiveness of CART therapy. Cell transfusion of cells memory T cells was superior to effector T cells thereby during the process of generation this type of population should be mainly achieved (McLellan and Ali Hosseini Rad 2019). However, there are mixed results on memory phenotype and efficacy of CAR T cells between studies and CAR constructs (Gomes-Silva and Ramos 2018).

Except the T lymphocytes, natural killer (NK) cells represent another subset of immune cells that can also be engineered to express chimeric antigen receptors, and thus be applied as a form of adoptive cell therapy for haematologic malignancies. NK cells are able to induce a different spectrum of cytokines, mainly IFN-γ, IL-3 and the granulocyte–macrophage colony-stimulating factor (GM-CSF). NK cells may not fully replace CART therapies, but they might become a form of complementary therapy. CART are being extensively used in clinical trials, with gaining the FDA approvals in recent years, while the CAR-NK construct has just entered the clinical trials market, with the first results published in 2020 (Glienke et al. 2015; Liu et al. 2020).

## Toxicity of CART

Even though clinical trials made with the adoptive transfer of CART have shown compelling results in the treatment of haematological malignancies, adoptive T-cell therapy is still limited by possible serious complications. The toxicities following CART infusion may be either immediate or delayed. The most common toxicity related to the CART therapy is the cytokine release syndrome (CRS). Another toxicity immediately following the CRS is neurotoxicity. It is correlated with neurological abnormalities, which untreated may become life-threatening. Hence, an enormous portion of CART research focuses on minimizing the toxicity of therapy (Bonifant et al. 2016).

CRS is the most common, adverse effect associated with CART therapy (Xu and Tang 2014). In most cases, the onset of CRS occurs within the first week of infusion and is connected with such factors as the type of malignancy or time of T-cell expansion peak. CRS is characterized by excessive activation of lymphocytes, dendritic cells and macrophages, as well as other compounds playing significant roles in immune response, Excessive stimulation of immune response results in the redundant release of inflammatory cytokines, including IFN-γ, and IFN-α, Il-1, IL-5, IL-6, IL-8, IL-10, IL-12, tumour necrosis factor α, monocyte chemotactic protein 1, macrophage inflammatory protein and GM-CSF (Maude et al. 2014). IL-6 is one of the major cytokines released in CRS and it crucial for the syndrome pathophysiology (Shimabukuro-Vornhagen et al. 2018). There have been various studies (Davila et al. 2014; Teachey et al. 2016; Frey 2017; Li et al. 2017; Murthy et al. 2019; Sievers et al. 2020) focusing on the causes of severe CRS development. CART-induced CRS in mice models showed that this type of toxicity is not delivered by direct mediation of CART-derived cytokines, but rather it is caused by IL-6, IL-1 and NO produced by recipient macrophages (Giavridis et al. 2018). Still, the detailed mechanism of CRS remains to be poorly defined (Wang and Han 2018). The cytokine profile observed during the CRS development is similar to the one of the macrophage activation syndrome (MAS). This may lead to misdiagnosis and misinterpreting the CRS symptoms as MAS. The first, visible manifestation of CRS is high fever, developing in most patients up to 40 °C or more (Li et al. 2017). The CRS intensity is defined as mild and severe types, referring to different symptoms of varying intensity. In mild cases, grade 1 and 2 CRS presents with flu-like, moderate symptoms and can be self-limiting (Zhao et al. 2019). Severe CRS of grade 3 and higher, due to threatening symptoms such as hypoxia, hypotension, may result in organ toxicity (Bonifant et al. 2016; Yeku and Brentjens 2016; Zhu et al. 2016; Giavridis et al. 2018). Patients affected with severe CRS might need mechanical ventilation to support their breathing, otherwise, the insufficient respiration may become life-threatening (Giavridis et al. 2018). In severe CRS ferritin and C-reactive protein achieve high levels in the blood. Developing grade 4 CRS may also result in delayed recovery from haematopoietic malignancies after the implemented therapy (Turtle et al. 2016). There are many factors that may lead to developing severe CRS. High tumour burden is a predisposing factor presented in a vast majority of patients affected with severe CRS. It is proven, that in high tumour burden-ALL patients, CRS occurs more frequently and in a more severe form. Severe thrombocytopenia is another disease affecting in high risk of developing CRS. Due to lower platelet level and thus, low levels of Ang-1, patients might be liable to endothelial activation (Li et al. 2017). Treatment implementation is advised in managing the symptoms of CRS induced by CART therapy. The first-line therapy applied in patients with CRS involves humanized monoclonal anti IL-6 receptor antibody—tocilizumab. Application of this particular antibody has predominantly resulted in overall clinical improvement, including decrease of temperature in case of fever, or reduction of heart rate in cardiovascular disfunction (for instance tachycardia) among patients. The main advantage of tocilizumab application is the fact that it does not affect the levels of CART cells in peripheral blood. Apart from tocilizumab, CRS management incorporates the implementation of corticosteroids. Some of severe CRS cases require high doses of corticosteroids. These are often the cases unresponsive to tocilizumab and characterized by life threatening symptoms. Unfortunately, the application of corticosteroids leads to decreased levels of CART cells in patients’ system. It has been proven that the outcome of sCRS management may affect the treatment results so it is imperative to identify the type of CRS properly and to implement appropriate medical treatment (Davila et al. 2014). The highest efficacy of corticosteroids is observed in the patients with low grade CRS. However, use of corticosteroids may hinder the efficacy of CART therapy (Patel et al. 2014). Increased cytokine levels play an important role in CRS and CRS-related toxicities. Because of that, numerous antagonists of cytokines have been applied to treat patients undergoing CART-related CRS (Xu and Tang 2014). However, blockade of IL-6 receptor may require further treatment using high doses of corticosteroids. Recent research showed that IL-1 receptor antagonists protect from severe CRS without compromising antitumour efficacy (Giavridis et al. 2018).

Another prominent toxicity occurring in patients undergoing CART therapy is neurotoxicity, which leads to neurological abnormalities including immune effector cell-associated neurotoxicity syndrome (ICANS). It usually occurs within the first week from the treatment implementation due to its life-threatening aftereffects (Wang and Han 2018). Typically, it is manifested as a toxic encephalopathy with aphasia, confusion, word-finding difficulty. However, in severe cases, it can be progressed in coma, motor weakness, cerebral edema. Death cases triggered by neurotoxicity related effects have also been reported (Locke et al. 2019). The exact mechanism of CART associated neurological events is not well known. There are some speculations about particular mechanisms that may be involved in its development. A recent study has shown a remarkable function of endothelial cells activation in the central nervous system neurotoxicity early-inducing mechanisms. According to the results, severe neurotoxicity may be associated with coagulopathy or vascular leak (Mackall and Miklos 2017). Similar to CRS cytokines, chemokines as well as CART expansion degree could be associated with severity of this toxicity (Neelapu 2019). Although neurotoxicity is a common adverse effect, severe cases of neural events are decreasing with the improvement of administered CART. At this point, the neural effects are easily reversible with proper medication and rarely become life threatening (Santomasso et al. 2018). The management of ICANS depends on the severity of symptoms. However, considering the issue of medication required for ICANS treatment, the majority of applied drugs are corticosteroids (low-dose during mild ICANS—dexamethasone or high dose during severe ICANS—methylprednisolone) and antiepileptics (levetiracetam) (Garcia Borrega et al. 2019).

The mechanism of action of CART is to recognize antigens present on malignant cells, which leads to their eradication. The ideal therapeutic agent would be restricted only to the malignant cells. However, currently developed modifications of CART target antigens that are present not only on the disease-associated cells, but also on healthy cells. Because of that, infusions with modified T cells may result in the immune system attacking healthy tissues and causing adverse effects in patients. The effects are not restricted to the system of the targeted malignancy and may occur in cardiovascular or pulmonary systems, among others. One of the most common effects caused by the on-target/off-tumour mechanism is B-cell aplasia, observed mainly in response to the CD19-targeting CART therapy. Another common occurrence in response to the CART therapy are cytopenias, including anemia, leukopenia, neutropenia, lymphopenia and thrombocytopenia. These effects are often actually associated with the conditioning therapy prior to the CART infusion, although they have also been reported in patients who did not receive conditioning chemotherapy (Bonifant et al. 2016; Yáñez et al. 2019; Neelapu 2019).

Another factor contributing to the toxicity of CART-based therapy is the source of antigen-recognizing domains in the modified cells. The majority of clinical trials acquire the mouse antibodies, which carries a potential for inducing an allergic reaction towards a foreign immunogenic compound. To lower the risk of causing severe anaphylaxis, introducing humanized scFVs rather than murine-based fragments into the engineered T-cell is advised (Casucci et al. 2015).

## CART Therapy in Haematological Malignancies

CART have revolutionized immunotherapy and remarkable responses achieved in clinical trials have lead to their increasing clinical use and first FDA approvals. Currently, adoptive cell immunotherapy is one of the most promising approaches of cancer therapy and the optimistic results in clinical trials have triggered the pharmaceutical industry to invest in this particular form of cancer conditioning. Since modified CART are derived from the immune system, they are most effective in treating haematological malignancies, including ALL, chronic lymphocytic leukaemia (CLL), diffuse large B-cell lymphoma (DLBCL) and multiple myeloma (MM) (Table 1). The first step in implementing CART in the treatment of those malignancies is to find a viable antigen target. Currently, most of the CART-based clinical trials use the CD19 antigen as a therapeutic target. As of today, there are two CART therapies approved by the FDA, Tisagenlecleucel and Axicabtagene ciloleucel, which are anti-CD19-CART agents used to treat ALL and DLBCL. Although CART therapy has shown promising results in patients with haematological malignancies, it still poses several risks that may lead to pathophysiological events. The most common adverse effects are ongoing CRS, neurotoxicity and B-cell aplasia (Brentjens et al. 2011). Safety analysis of studies encompassing the use of CART demonstrated the presence of advert events in majority of patients undergoing this therapy. Fortunately, those side effects are characterized by acceptable toxicity level and the tendency to be short termed (Lee et al. 2015).

**Table 1:** Selected clinical trials for CARTs in haematological malignancies

Study	Phase	Condition or disease included in data analysis	CART generation	Costimulatory domain	Target molecule	Outcome (*n*/*n* infused)	References
NCT01593696	I	ALL	2nd	CD28	CD19	CR: 70% (14/20)	Lee et al. (2015)
NCT01044069	I	ALL	2nd	CD28	CD19	CR: 83% (44/53)	Park et al. (2018)
NCT02028455 (PLAT-02)	I/II	ALL	2nd	4-1BB	CD19	CR: 93% (40/43)	Gardner et al. (2017)
NCT02772198	I/II	ALL	2nd	CD28	CD19	CR: 90% (18/20)	Jacoby et al. (2018)
NCT02435849 (ELIANA)	II	ALL	2nd	4-1BB	CD19	CR: 81% (61/75); FDA approval for ALL	Maude et al. (2018)
NCT01029366	I	CLL	2nd	4-1BB	CD19	OR: 57% (8/14; 4 CR and 4 PR)	Porter et al. (2015)
NCT01416974	I	CLL	2nd	CD28	CD19	OR: 38% (3/8; 2 CR and 1 PR)	Geyer et al. (2018)
NCT01865617	I/II	CLL	2nd	4-1BB	CD19	OR: 74% (14/19; 4 CR and 10 PR)	Turtle et al. (2017)
NCT03331198 (TRANSCEND CLL 004)	I/II	CLL	2nd	4-1BB	CD19	OR: 87% (13/15; 7 CR and 8 PR)	Siddiqi et al. (2019)
NCT00924326	I	DLBCL	2nd	CD28	CD19	OR: 68% (13/19; 9 CR and 4 PR)	Kochenderfer et al. (2017)
NCT02631044 (TRANSCEND NHL 001)	I	DLBCL	2nd	4-1BB	CD19	OR: 68% (89/131; 64 CR and 25 PR)	Abramson et al. (2020)
NCT02348216 (ZUMA-1)	I/II	DLBCL, PMBCL, tFL	2nd	CD28	CD19	OR: 83% (84/101; 59 CR and 25 PR); FDA approval for DLBCL	Viardot et al. (2019)
NCT02445248 (JULIET)	II	DLBCL	2nd	4-1BB	CD19	OR: 52% (48/93; 37 CR and 11 PR); FDA approval for DLBCL	Schuster et al. (2018)
NCT02215967	I	MM	2nd	CD28	BCMA	OR: 81% (13/16; 2 sCR, 8 VGPR and 3 PR)	Brudno et al. 2018
NCT02658929	I	MM	2nd	4-1BB	BCMA	OR: 85% (28/33; 15 CR and 13 PR)	Raje et al. (2019)
NCT01886976	I/II	MM	2nd	4-1BB	CD138	OR: 80% (4/5; 3 SD	Guo et al. (2016)

*ALL* acute lymphoblastic leukemia, *CLL* chronic lymphocytic leukemia, *DLBCL* diffuse large B-cell lymphoma, *PMBCL* primary mediastinal B-cell lymphoma, *tFL* transformed follicular lymphoma, *MM* multiple myeloma, *OR* overall response, *CR* complete response, *sCR* stringent complete response, *PR* partial response, *VGPR* very good partial response, *SD* stable disease

## CART in ALL

ALL is a haematological malignancy characterized by a transformation and proliferation of lymphoid progenitor cells. Malignant cells are present in bone marrow, blood, and extramedullary sites. ALL affects both children and adults, but its incidence is most prevalent between ages 2 and 5, making children 80% of all patients diagnosed with this condition (Terwilliger and Abdul-Hay 2017). Although the 5-year survival with ALL has been steadily improving for children, reaching up to 90%, the survival rate for adults is estimated at about 40% (Paul et al. 2016). The prognosis for the relapsing and refractory subtype of ALL is much worse than for primary ALL, with long-term overall survival (OS) rates of 15–50% and currently available treatment options being unsatisfactory (Nguyen et al. 2008). Therefore, there is a need for developing new therapeutic approaches to treat ALL, especially after the disease relapse.

CART pose an effective new treatment for ALL, especially the CD19-targeting agents, as CD19 is present on B cells and thereby is a viable therapeutic target (Inaba et al. 2013; Vairy et al. 2018). CD19-CART therapy in pediatric and young adult ALL was the first CART treatment that received the FDA approval (Liu et al. 2017). Both global, and single-centre studies revealed the numerous durable remissions from ALL after application of CART therapy. The overall range of successive outcomes is included in between 67 and 93%, making the CART therapy the most efficient in relapsed and refractory types of ALL (Grupp et al. 2015; Lee et al. 2015).

Although ALL is mostly affecting children, the use of CART for this malignancy has been investigated in all age groups. The first successful administration of CART in therapy of ALL was reported in 2013, as first published results of phase I clinical trial (NCT01626495) assessing the safety and feasibility of anti-C19 CART in therapy of children with chemotherapy resistant or refractory CD19^+^ B-cell cancers. In the study, the CAR receptor was introduced on modified T cells via a lentiviral vector during manufacturing and the modified construct contained a 4-1BB costimulatory domain. Two child patients with relapsed and refractory pre-B-cell ALL received CD19-targeting modified CART, which resulted in CR in both cases and was ongoing in one of the patients for almost a year after treatment (Grupp et al. 2013).

Treatment of older children and young adults with relapsed or refractory ALL was investigated in a phase I dose-escalation clinical trial (NCT01593696). Patients under the age of 30 with relapsed or refractory ALL received infusions of CD19-targeting T cells with a CD28 domain. 60% of them achieved a minimal residual disease (MRD)-negative after the CART treatment. Since the usual follow-up step for MRD-negative remission in young patients with ALL is haematopoietic stem cell transplantation (HSCT), the majority of them underwent the transplant after achieving the remission and they remained leukaemia-free at the time of the median 10 month follow-up analysis. This study concluded that the CD19-CART therapy is not only a viable form of treatment, but also an effective approach to prepare patients with chemorefractory ALL to undergo HSCT (Lee et al. 2015).

Although this haematological malignancy mostly affects pediatric patients, there have also been clinical trials focusing on using CART cells to treat adult patients with relapsed or refractory ALL. In a phase I clinical trial (NCT01044069) 53 adult patients received the treatment with modified T cells, the median age of this group being 44 years. These patients previously received other extensive ALL-targeting therapies, as for 36 (68%) of these patients, the CART therapy was a third or later line of treatment. Some patients also previously received anti-CD19 treatment with blinatumomab, a bispecific T-cell engager targeting CD3 and CD19. The administered anti-CD19-CART treatment resulted in CR in 44 out of the 53 (83%) patients and the remission rate did not differ between groups of different therapeutic backgrounds. The results of this trial suggest that CART treatment may be an efficient option for patients who previously received other extensive regimens, including therapies focusing on the same antigens as ones recognized by CAR constructs (Park et al. 2018). Similar conclusions can be drawn from other clinical trials, in which patients with relapsed or refractory ALL received anti-CD19 agents before CART treatment, with even 90% of patients achieving complete remission (Gardner et al. 2017; Jacoby et al. 2018).

ALL was the first haematological malignancy to receive a CART-based FDA approval for therapy. The ELIANA (NCT02435849) phase II clinical trial was conducted to assess the effectiveness and safety of anti-CD19 CART therapy, using a developed therapeutic agent called tisagenlecleucel. This CAR construct is manufactured using a lentiviral vector and it contains a 4-1BB costimulatory domain. In this study, 75 pediatric and young adult patients with CD19^+^ relapsed and refractory ALL received tisagenlecleucel infusions and 96% of the patients had lymphodepleting chemotherapy prior to the treatment. With the primary endpoint of the study being the overall remission rate within 3 months, the successful implementation of therapy was noted in 81% of the patients, with 45 patients achieving CR and 16 patients having complete remission with incomplete haematologic recovery (Maude et al. 2018). The follow-up quality of life report of patients who received tisagenlecleucel in the ELIANA study suggested that benefits of CART treatment in CD19^+^ ALL overweight the risks of the therapy (Laetsch et al. 2019). Based on the results of the ELIANA study, tisagenlecleucel got FDA-approved in 2017 to treat young patients with relapsed or refractory ALL (Liu et al. 2017).

## CART in CLL

CLL is a haematological malignancy occurring in adults, characterized by a clonal proliferation of mature lymphocytes. The risk of developing CLL increases with age, with the median age of diagnosis being 70 years (Smith et al. 2011; Mewawalla and Nathan 2014). There is a widespread search for viable therapeutic agents that could improve CLL prognosis and increase the survival rate of patients. Although the immunodeficiency development in CLL pathogenesis poses CART-based therapy as more difficult to conduct due to problems with engineered T-cell expansion, there have been various studies focused on applying CART in clinical use for this type of leukaemia (Zhao et al. 2018).

The first reported use of CART therapy to treat CLL was a single patient case in 2010, who received CART infusion and achieved a CR in bone marrow after 23 days (Porter et al. 2011). After that, the use of CART on a bigger cohort was described in another report from the same phase I clinical trial (NCT01029366). The study used lentivirus-modified CART with a 4-1BB domain to achieve a therapeutic effect. 14 out of 23 (61%) patients received the modified T-cell infusions and the median age of the group was 66 years. All patients had a long history of previous treatments, mostly chemotherapy-based and at the time of CART therapy they had an active disease. At the follow-up, 8 out of 14 (57%) patients responded to the anti-CD-19-CAR-T treatment and the CR was noted in 4 out of 14 (29%) infused (Grupp et al. 2015; Porter et al. 2015).

There have also been trials focused on using CART not just as an main form of treatment, but also as a consolidation therapy is standard methods are not sufficient enough to control the disease progression. One of these trials enrolled patients with CLL who did not receive previous treatment. The study protocol included initial chemoimmunotherapy with pentostatin, cyclophosphamide and rituximab and CART infusions as a form of consolidative therapy for those patients, who did not achieve complete remission after chemotherapy. CART used in this trial had a CD28-based a costimulatory domain. Responses to the CART therapy were noted in three out of eight (38%) patients, with two (25%) patients achieving clinical CR and one (13%) achieving partial remission of disease in the bone marrow (Geyer et al. 2018).

Other trials considered CART therapy as a next line of treatment for heavily pre-treated patients with CLL. Patients enrolled in these trials received previously unsuccessful treatments with therapeutic agents commonly used in treating CLL, such as ibrutinib. CART have proven to be effective in patients who either did not respond to standard therapies or developed resistance over time (Turtle et al. 2017; Siddiqi et al. 2019).

Current studies on CART in CLL are focusing on significant factors that may improve their effective response. Along with progressive investigations, novel strategies for CLL therapy have been suggested. Some of those are considering a change of the target antigen. Although CD19 remains an attractive target, due to its presence on malignant B cells, the better aims for CLL therapy, such as CD20 are still investigated (C. Hosing, P. Keabriaei 2014).

## CART in DLBCL

DLBCL is the most common type of non-Hodgkin’s lymphoma in adults and it makes up approximately 30–40% of all lymphoma cases worldwide (Li et al. 2018). It is characterized as a neoplasm of large B cells arranged in a diffused pattern. For cells to be defined as large, they have to be bigger that the nuclei of benign histiocytes present in the same area. The median age of DLBCL patients at the time of diagnosis is 70 years. Current 5-year OS for DLBCL is 60–70% after standard therapy, but median OS for untreated DLBCL patients does not reach one year. Despite the fact that the majority of patients can respond well to the first-line chemotherapy, acquiring the primary refractory disease or relapsing is not rare (Rovira et al. 2015; Li et al. 2018). As malignant cells express many B-cell antigens, including CD19, CD20 and CD22, they could potentially become successful targets for CART therapy, especially for patients that could not benefit from commonly used treatments (Smith et al. 2011; Rovira et al. 2015; Li et al. 2018).

Most clinical trials that focus on using CART in the therapy of DLBCL include multiple types of other B-cell lymphomas in the studies, such as mantle cell lymphoma (MCL), follicular lymphoma (FL), primary mediastinal B-cell lymphoma (PMBCL), with DLBCL being one of the trial cohorts (Kochenderfer et al. 2017; Abramson et al. 2018a, b, 2020; Locke et al. 2019).

Study designs and protocols used for treating B-cell lymphomas are similar to those of the trials focusing on ALL and CLL, with introducing lymphodepletion chemotherapy prior to the CART infusion. A small phase I clinical trial (NCT00924326) aimed to assess the efficacy and safety of CART therapy in patients with B-cell lymphomas, including MCL, FL and DLBCL. Over a half of treated DLBCBL patients had a chemotherapy-refractory form of lymphoma and other patients had DLBCL that relapsed after a maximum of ten months after receiving autologous stem cell transplantations as the most recent treatment. The overall remission among DLBCL patients was 68%, out of which 47% achieved CR and 21% achieved partial remission. The study also measured the levels of IL-15, as it is known to induce the proliferation of T lymphocytes. Biochemical analysis showed that the expression of IL-15 is associated with the effectiveness of therapy and its levels are higher in patients that had a stronger response to the CART treatment, as blood IL-15 levels were higher in patients that achieved disease remission (Kochenderfer et al. 2017).

Another trials also showed promising results of CART treatment for patients with DLBCL. In the TRANSCEND NHL 001 phase I clinical trial (NCT02631044) patients received a novel CD19-CART agent called lisocabtagene maraleucel, which uses 4-1BB as a costimulatory domain. The study included patients with relapsed and refractory aggressive non-Hodgkin lymphomas, including DLBCL, PMBCL, FL and MCL. In the cohort of 131 patients with not otherwise specified DLBCL, 89 out of 131 (73%) patients responded to the therapy, with 64 (49%) of them achieving complete remission (Abramson et al. 2018a, b, 2020).

Another investigated anti-CD19 CART agent in DLBCL treatment is tisagenlecleucel, also used in ALL therapy. In the phase II JULIET clinical trial (NCT02445248) a total of 93 patients received CART infusions and were included in the study evaluation. The trial enrolled adult patients with relapsed or refractory DLBCL that had a poor prognosis. The complete response to the therapy was achieved in 40% of patients and the remaining 12% demonstrated partial response. The updated results of JULIET revealed that CART therapy can contribute to durable remissions among patients with relapsed or refractory DLBCL. One of the secondary endpoints was OS and it was met due to persistence and efficacy of infused CART (Schuster et al. 2018).

One of main trials for CART in DLBCL treatment was ZUMA-1. It was a phase I/II clinical trial that enrolled patients with refractory large B-cell lymphomas, including DLBCL, PMBCL and transformed FL. Patients received axicabtagene ciloleucel, an autologous anti-CD19 CART therapy. Across 2 trial phases, a total of 101 patients both received the modified T-cell therapy and were assessable for study results analysis at a median long-term follow-up of 27.1 months. The follow-up concluded that 84 out of 101 (83%) patients had an objective response, with 59 (58%) patients achieving complete response and 25 (25%) achieving partial response. Although the study included different sub-types of refractory B-cell lymphomas, the treatment outcomes were similar across all groups with different malignancies. The study also showed durable responses achieved even with a single infusion of axicabtagene ciloleucel. The remission lasted for more than 2 years and did not require consolidation therapy (Locke et al. 2019). Based on the results of the ZUMA study, axicabtagene ciloleucel received the FDA approval in 2017 for the treatment of refractory and relapsed DLBCL (Viardot et al. 2019).

After the FDA approval of axicabtagene ciloleucel, there has been a rise of facilities interested in using the CD19-targeting CART therapy to treat the patients with CART in real clinical practice and outside of clinical trials. There were two studies focused on the use of anti-CD19-CART to replicate the results of ZUMA-1 study to treat patients with refractory DLBCL (Chavez et al. 2019). Both non-clinical real-world studies included patients with DLBCL with a median age of over 60 years. Interestingly, even though about half of the patients included in these studies would not fill the ZUMA-1 eligibility criteria, in both cases the therapy efficiency was similar to the one described in the clinical trial (Jacobson et al. 2018; Nastoupil et al. 2018; Herrera 2019; Chavez et al. 2019) These studies show that the effects of CART therapy are not limited only to the highly restricted environment and conditions of clinical trials. CART, despite requiring patient personalization, might become more accessible with their further development (Chavez et al. 2019).

## CART in Multiple Myeloma

MM is a haematological malignancy characterized by an expansion of malignant plasma cells that accumulate in the bone marrow and overproduce a monoclonal protein. This disease occurs in adults, with patients older than 65 making up 85% of all MM cases. The occurrence of MM is high, as it accounts for approximately 10% of all haematological malignancies (Michels and Petersen 2017). Although the OS in patients with MM is constantly being improved, the disease itself remains without permanent treatment. Despite high numbers of received therapies, all MM patients eventually relapse. This is often associated with acquiring resistance to previous therapies, thus limiting the therapeutic options to achieve another remission. Hence, different MM-targeting therapies are being developed and CART pose a viable option to treat patients with a MM relapse. The main promising target for CART in MM treatment is the B-cell maturation antigen (BCMA) which is expressed on the surface of plasma cells (Rajkumar 2019; Huang et al. 2020).

There have been multiple clinical trials focused on using CART to target BMCA molecules in patients with MM (Ali et al. 2016; Brudno et al. 2018; Mailankody et al. 2018; Shah et al. 2018; Cohen et al. 2019; Raje et al. 2019).

The first study aiming to assess the safety and efficacy of CART targeting BCMA in previously treated patients with MM enrolled 24 patients with heavily pre-treated relapsed and refractory MM. Patients that received the highest doses of modified cells had the strongest response to the therapy, but the adverse effects were the most severe in that group (Ali et al. 2016; Brudno et al. 2018).

Another clinical trial also targeted malignant cells in MM patients. The modified CART drug with a 4-1BB domain, called bb2121, was administered as a single infusion. All patients who received the modified cells had a long history of previous treatments. While the implemented CART treatment was targeting BMCA, the response to the therapy seemed to be independent of its expression level (Raje et al. 2019). Similar CAR-BCMA-T cell drug, bb21217, was tested in a phase I CRB-402 clinical trial (NCT03274219) for patients with relapsed MM, in which six out of seven (86%) patients showed a response to the treatment. The CART drug bb21217 is based off bb2121, as it also uses the 4-1BB costimulatory motif. The difference between these drugs is based on the manufacturing process, as bb21217 is exposed to phosphoinositide 3 kinase inhibitor bb007 during its synthesis, which increases the number of T cells with a memory-like phenotype obtained in the process (Shah et al. 2018).

Other therapeutic targets have also been considered in the CART-based treatment of MM. One of them is CD138, a molecule highly expressed on MM cells and involved in the development and proliferation of malignant cells. Because of that, CD138 seems to be a viable target for novel immunotherapy-based treatments (Tassone et al. 2004; Polson and Sliwkowski 2009).

A small phase I/II clinical trial (NCT01886976) was designed to assess the safety and feasibility of anti-CD138-CART treatment, along with the duration of in vivo survival of modified T cells. In response to the therapy, in four out of five (80%) patients the myeloma regression was noted, three patients had stable disease for over 3 months and one patient later advanced to plasma cell leukaemia (Guo et al. 2016).

Although the CD19-directed therapy gains most attention in the treatment of ALL and DLBCL, especially due to the FDA approval of tisagenlecleucel and axicabtagene ciloleucel, there have been studies dedicated to using this compound in the treatment of relapsed and refractory MM. CD19 is not a common antigen present in MM as its expression on plasma cells is usually close to none, but there is a minor subset of MM cell that may express it at low levels. It is suggested that this subset of cells are precursors of malignant plasma cells, thus targeting them could be beneficial in preventing the expansion of the disease. However, it is hypothesized that CD19^+^ MM cells can only be targeted after the eradication of non-CD19 MM cells using chemotherapy. Nonetheless, there have been several clinical trials focused on using anti-CD19-CART in the therapy of MM (Garfall et al. 2015; Sidana and Shah 2019).

One of them was a phase I clinical trial (NCT02135406) that focused on the safety, tolerability and engraftment potential of modified CD19-targeting CART in patients with MM after the melphalan treatment and autologous stem cell transplantation. The patients progressed within a year post-transplantation. Ten patients received the cell infusions and were included in the study analysis. At 100 days after the T-cell therapy, eight out of ten (80%) patients had at least a partial response (Garfall et al. 2018).

While there is a search for different therapeutic targets for CART in MM treatment, BMCA remains the most often used antigen for this form of therapy. However, exploring other possible targets is important for further development of CART use in myeloma treatment (Goldschmidt et al. 2018).

## Limitations and Challenges of CART Therapy

CART therapy is a promising approach to treat malignancies, but there are some limitations and questions for this type of treatment. The first factor that limits the use of CART therapy is its cost and availability. The process of generating modified T cells is highly personalized, as the constructs have to be produced specifically from the patient’s immune cells. In contrast to other existing immunotherapy-based approaches, such as immune checkpoint inhibitors, CART are not universal and cannot be mass produced. This reflects in high therapy costs and a limited number of facilities that can prepare and supervise the therapy. Genetic engineering of T cells requires advanced technologies using either viral vectors or other tools of gene editing, which may not be accessible in smaller laboratories. Patients receiving CART therapy also have to be monitored in facilities fully equipped to provide a highly controlled and sterile environment to lower the risk of infections (Hettle et al. 2017).

There is also a risk of developing resistance to CART therapy in response to prolonged exposure to the modified cells especially in patients with relapse of ALL with negative expression of CD19 or with splice variant of CD19 without exon 2 that is recognized by scFv of CAR-T cells (Jacoby et al. 2018). The barriers to durable CART-dependent remissions are characterized by developed complex mechanisms in which they can lower, or even block the effective potency of adoptive T-cell therapy. The most common cause of unsuccessful therapy is the CART failure which can be caused by multiple factors, such as insufficient of expansion or limited persistence of engineered cells in vivo. Another cause of resistance to therapy is the antigen loss or its down-regulation. In some cases, the disease relapse results in malignant cells no longer expressing the antigen targeted by the first course of CART therapy (Shah and Fry 2019).

The clinical trial enrollment process requires potential patients to fill multiple eligibility criteria. During enrollment, some patients cannot qualify as subjects for the CART therapy. One of the main reasons lies in the immunity issue. Apart from the presence of targeted malignancy, patients are required to be in a relatively good health condition to undergo therapy. Since most protocols of modified T-cell infusions include preparative lymphodepletion chemotherapy, patients with immunodeficiencies are at risk of highly endangering their lives with the pre-treatment alone. Moreover, being in a state of immune deficiency right before the T-cell administration exposes vulnerable patients to infections. Most patients undergoing CART therapy present poor immune function, related both with the prior cytotoxic treatment and the CART therapy itself. Reports state, however, that the immunodeficiency-related complication is similar to that of different cancer therapies, including chemoimmunotherapies (Hill et al. 2018; Smith et al. 2019).

## Conclusions

Every step of CART construction performed in vitro is significant for maintaining proper architectural properties of the construct and thus, it affects CART mechanism of action in vivo. The results of clinical trials performed in haematooncology provided promising effectiveness of CART in ALL, DLBCL, CLL, and MM. However, in some patients, there is also a risk of developing resistance to CART therapy in response to prolonged exposure to the modified cells. Thereby there are still a lot of questions that are not resolved and require more observations to improve the effectiveness of this therapy and limit toxicities.

## Abbrevations

CAR: Chimeric antigen receptor
CART: CAR-T cell
ALL: Acute lymphoblastic leukaemia
DLBCL: Diffuse large B-cell lymphoma
CLL: Chronic lymphocytic leukaemia
MM: Multiple myeloma
TCR: T-cell receptor
scFv: Single-chain variable fragment
MHC: Major histocompatibility complex
ITAM: Immunoreceptor tyrosine-based activation motif
IL: Interleukin
ICOS: Inducible T-cell co-stimulator
NFAT: Nuclear factor of activated T cells
FDA: Food and Drug Administration
CD: Cluster of differentiation
NK: Natural killer
GM-CSF: Granulocyte–macrophage colony-stimulating factor
CRS: Cytokine release syndrome
IFN: Interferon
MAS: Macrophage activation syndrome
ICANS: Immune effector cell-associated neurotoxicity syndrome
OS: Overall survival
CR: Complete remission
MRD: Minimal residual disease
HSCT: Haematopoietic stem cell transplantation
MCL: Mantle cell lymphoma
FL: Follicular lymphoma
PMBCL: Primary mediastinal B-cell lymphoma
BCMA: B-cell maturation antigen
